# Categorization of drivers of change for emerging food safety risks

**DOI:** 10.1016/j.crfs.2025.101098

**Published:** 2025-06-01

**Authors:** N.M.C. Hommels, M.C.M. Mourits, M. Focker, H.J. van der Fels-Klerx

**Affiliations:** a Business Economics Group, Wageningen University and Research, Wageningen, the Netherlands; b Wageningen Food Safety Research, Wageningen University and Research, Wageningen, the Netherlands

**Keywords:** Multicriteria analysis, Emerging food risk, Food safety management, Pathogens, Contaminants

## Abstract

The emergence of food safety risks can be influenced by various causes, also known as drivers of change. Understanding the characteristics related to the manageability of these drivers and the health impact of their associated hazards is critical for effective food safety management and resource allocation. This study aims to categorize drivers of change for known food safety hazards based on their impact on human health and their manageability. Identified drivers were categorized and ranked through an expert survey and a PROMETHEE multi-criteria analysis. The weighted performance criteria are controllability and volatility of the driver, and likelihood and severity of the associated hazards.

Results show that the severity of associated hazards is the most important criterion, while volatility of the driver is considered least important by experts. All drivers of change were ranked based on their potential impact on food safety and categorized in a driver matrix with four categories. Categorization is based on the combined effect of expected negative health impact (low or high) and manageability (difficult or easy). The four categories are: “monitor and adapt” (low, difficult), “analyze and optimize” (low, easy), “leverage and innovate” (high, easy), and finally “strategize and endure” (high, difficult).

Two drivers—environmental contamination and geopolitical conflict—are categorized as difficult to manage and associated with highly likely and severe health consequences. These drivers represent critical challenges requiring long-term strategic efforts. Six drivers are highly manageable (low health impact: legislation, policies and governance, technologies in food production, food processing technologies; high impact: management of natural resources, bioprocesses, supply chain) and could be leveraged to influence food safety hazards. The results are shown to be robust against changes in criteria weighting.

These findings can support decision makers in shifting focus toward manageable drivers and tailoring strategies by driver category. For effective action, it is essential to understand the specific risks and benefits of each driver and the burden of their associated hazards. Future work should explore the top-ranked drivers and integrate these insights into holistic food safety management strategies.

## Highlights

• Severity of associated hazard is the most important criteria in ranking drivers.
• Drivers of change are categorized based on health impact and manageability.
• Custom management strategies are introduced per driver category.
• Geopolitical instability and environmental contamination are critical challenges.

## Graphical abstract

**Figure:**
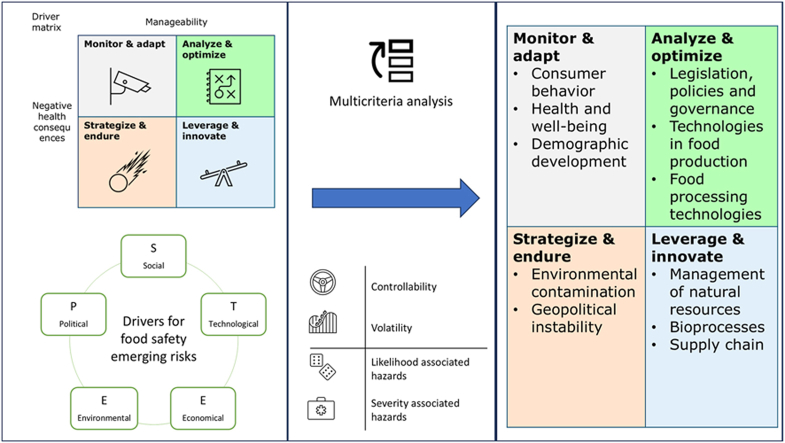

## 1 Introduction

Our connected and industrialized food system is prone to emerging food safety risks (Chammem et al., 2018; van de Brug et al., 2014). An emerging risk related to food safety (i.e. an emerging food safety risk) is defined by the European Food Safety Authority (EFSA) as a risk resulting from a newly identified hazard (a), or from an unexpected new or increased exposure (b) and/or susceptibility (c) to a known hazard (Ana et al., 2020; Gkrintzali et al., 2023; EFSA 2020; Costa et al., 2017). Emerging risks can have strong negative effects on food security and food safety (Fones et al., 2020; Gomez-Zavaglia et al., 2020). Therefore, in Europe, such risks are under constant observation, such as by an EFSA exchange network (EFSA 2020; Gkrintzali et al., 2023).

Drivers of change can lead to the emergence of food safety risks. A driver of change is an external or internal force affecting the food system which can influence directly or indirectly and shape the food system in a positive or negative manner (EFSA, 2010; Zurek et al., 2022). Understanding the drivers of change influencing emerging risks is crucial for effective food safety management (World Health Organization (World Health Organization, 2022). Insights into drivers and their specific characteristics will help decision and policy makers to adapt food safety management to a more tailored approach for more effective resource allocation and allow for a holistic food safety management system and reduce the potential negative effects of emerging food safety risks (Farkas et al., 2023; Kleter and Marvin, 2009; Marvin et al., 2009).

In a prior study, Hommels et al. (2025) identified the key drivers of change impacting food safety in Europe and westernized food systems, utilizing the STEEP framework (Grima et al., 2020) to consider social, cultural, technological, economic, environmental, and political factors. However, to date, health impact and manageability of the drivers have not been investigated and there is a limited understanding of how to manage drivers. The aim of this study is to investigate the potential negative health impacts related to drivers of change and the manageability of these drivers. The underlying aim is to perform a ranking and subsequent categorization in health impact – manageability driver matrix quadrants (EFSA, 2010). Results contribute to strategic and effective food safety management from a holistic perspective while allowing for tailored response to emerging food safety risks (Chammem et al., 2018; Garcia et al., 2020).

## 2 Methodology

### 2.1 Overview

This study focuses on drivers of change of emerging risks from known hazards, as investigated by Hommels et al. (2025). The drivers of change are defined as: “A driver may act as modifiers of effect on the onset of emerging food safety risks, namely they can either amplify or attenuate the magnitude or frequency of risks arising from various sources” (EFSA, 2010). In this study, first, the drivers of change are ranked using expert opinion. Second, they are categorized and assessed using a questionnaire based on multiple criteria analysis (MCA). Ethical clearance for this study is obtained from the WUR Research Ethics Committee for review of non-medical studies (2023–044).

### 2.2 Multicriteria analysis

The list of 11 drivers of change for emerging risks arising from known hazards, as identified by Hommels et al. (2025), was used as the starting point for this study. The list includes.

- social drivers: consumer behavior, demographic development, health and wellbeing.
- technological drivers: technologies in food production, technologies in food processing.
- economic driver: supply chain.
- environmental drivers: environmental contamination, management of (natural) resources, bioprocesses; and
- political drivers: legislation, policies and governance, and finally geopolitical instability.

These drivers are ranked using an expert respondent survey designed based on MCA, specifically the utility based PROMETHEE method. MCA provides a systematic approach to evaluate and rank alternatives (in this case the drivers) against multiple criteria. The benefit of using this method is the transparency, simplification of a multifaceted problem and allowing for multiple viewpoints (Ali et al., 2022; Fazil et al., 2008). A couple of studies have used MCA in the food safety domain previously, focusing on identifying relevant hazards or food safety measures, or tallying risks versus benefits (Ali et al., 2022; Banach et al., 2021; Garre et al., 2020; Van der Fels-Klerx et al., 2018).

Following the general outline of an MCA, a problem definition with various alternatives is presented to the experts involved (Ali et al., 2022). Instead of alternatives being scored on criteria and overall preference, in this study, the drivers of change are scored on a set of criteria. The criteria differentiate the drivers from one another and present critical attributes used for decision making or ranking (Fazil et al., 2008). The defined criteria to rank the identified drivers of change are based on literature (Béné et al., 2016; Lindqvist et al., 2020; Mu et al., 2021; Pimm et al., 2019; Van der Fels-Klerx et al., 2018). The criteria are: 1) volatility of the driver, 2) controllability of the driver, 3) likelihood and 4) severity of associated hazards. Volatility is defined as the relative degree of variation or fluctuation in the driver of change over time. Controllability refers to the extent to which a driver and its impact can be controlled over time. Likelihood indicates the chance that the associated hazard will (continue to) affect food systems now and in the close future. The severity refers to the extent of negative health effects caused by the hazards associated to the particular driver of change.

Preference weights - which indicate the relative importance of each of the four - are provided by the experts. Finally, the same experts are asked to score each driver based on the four criteria (see 2.3.2). A measure of overall score, based on the weights given by the experts and the PROMETHEE preference function, results in a final group ranking.

#### 2.2.1 Experts

In this study, the same group of experts as in Hommels et al. (2025) is used which includes food safety experts associated with FoodSafeR, a European project with experts from industry, academia and regulatory agencies (FoodSafeR consortium, 2022). Briefly, 51 experts completed the survey, of which 68.6 % live or work in the European Union. 72.6 % reported themselves to be very or even excellently knowledgeable. Of the respondents 16.3 % work in industry, 26.5 % work at a governmental institute, 22.4 % work at a research institute and 32.7 % work at university. The expert survey is conducted via Qualtrics (*Qualtrics*, Provo, UT) (Qualtrics, 2023).

#### 2.2.2 Criteria scores

By means of a questionnaire, experts are asked to score each driver of change for each of the four criteria, using a five-point Likert scale from “Not at all [criteria]” (1) to “Extremely [criteria]” (5) important. Instead of a neutral midpoint in the scale, as suggested by Ali et al. (2022), “Moderately [criteria]” (3) is used to eliminate the misuse of a neutral midpoint which has been found to be used a dumping ground of answers not corresponding to other scale points (Chyung et al., 2017).

#### 2.2.3 Criteria weights

Besides scoring the drivers per criterion, the experts are also asked to divide 100 points over the four criteria to indicate their relative importance weight. This weight represents the relative importance for prioritizing management efforts for individual drivers. This method is based on the WASPAS method which has a high accuracy of measurement (Garre et al., 2020; Zavadskas et al., 2012). The retrieved weights and driver scores are used as inputs for the PROMETHEE calculation of the driver ranking.

### 2.3 Ranking of drivers of change

The relative importance weights for the criteria and the scoring of the drivers, from the experts, are used to calculate critical ranking with the Visual PROMETHEE-GAIA software (Version 1.4, Academic Edition, B. Mareschal, Bruxelles, Belgium). In PROMETHEE, a preference function is used to perform pairwise comparisons under specifically defined preference and indifference thresholds. These thresholds indicate the points at which the difference in score leads to one driver being ranked higher than another driver. An indifference threshold is the point before which the difference between two drivers is irrelevant. The preference threshold is the point after which differences between drivers are relevant (Fazil et al., 2008). In this study, the level preference function is used with a preference and indifference threshold of 0. Setting the preference and indifference threshold to the same Likert score in a level preference function, gives a U shape preference function as previously used in the food safety research (Ali et al., 2022; Fazil et al., 2008).

#### 2.3.1 Optimal direction of criteria

For three criteria, the optimal direction is to maximize the score are volatility of the driver, likelihood and severity of associated hazards. In other words, to find the most pressing driver of change, which corresponds to the highest rank, the criteria are considered of higher rank if the score is larger (i.e. the direction towards maximize). For one criterium, i.e. controllability of the driver, the optimal direction is to minimize the score. Considering a higher controllability equals a more manageable driver, so the lowest score is highest in the critical ranking.

#### 2.3.2 Overall ranking

The overall ranking of drivers is determined by calculating a net outranking flow for each driver by summing the degree to which a driver outranks other drivers (positive flows) and the degree to which it is outranked by other drivers (negative flows) given the evaluated four criteria. The net flow of a driver provides an approximate measure of the overall critical pressure on the food system according to expert preferences, with higher net flows indicating more critical drivers. Based on the netflow results a critical ranking is established, where the top drivers are the most pressing on the food system.

### 2.4 Sensitivity analyses

A sensitivity analysis is performed to reflect on the robustness of the overall ranking. First, a separate PROMETHEE per subset of experts is performed. The subsets are created based on the expert's work environment, and include the following 5 groups: a food company, a governmental institute, a research institute, academia, or retirement. From the analysis per subset of experts, it can be seen how the results are affected by the background of the experts. Finally, the weights of the criteria which affect the netflows are changed, by varying their value from 0 to 100. In theory, a criterion could have a relative weight between 0 and 100, however these extreme values are unlikely as the 100 points have to be distributed over all four criteria by a respondent. The effect of changes in criteria weight is presented in visual stability plots, illustrating how the ranking of drivers shifts, as indicated by an intersection in the plot.

### 2.5 Categorization management opportunities of drivers of change

To capture the characteristics of the management opportunities of the evaluated drivers of change, a health impact – manageability matrix is created (Matrix Table 1), comparable to the risk matrix by Yu et al. (2024). It is further based on commonly used business science strategic decision-making systems SWOT and TOWS. A SWOT analysis can determine strengths and weaknesses, opportunities and threats, and TOWS is a strategic action matrix of the SWOT analysis (Maity et al., 2023). Combining multicriteria decision methodologies with SWOT can allow for actionable holistic insights (Kajanus et al., 2012).

**Table 1:** Netflow results from complete expert responses for the 11 main drivers.

Driver	Positive flow	Negative flow	Netflow	Rank
Environmental contamination	0.89	0.09	0.80	1
Geopolitical instability	0.78	0.19	0.58	2
Bioprocesses	0.75	0.25	0.50	3
Management of natural resources	0.57	0.39	0.17	4
Supply chain	0.55	0.42	0.13	5
Demographic development	0.51	0.46	0.05	6
Consumer behavior	0.43	0.54	−0.11	7
Health and well-being	0.38	0.57	−0.19	8
Food processing technologies	0.21	0.73	−0.52	9
Technologies in food production	0.15	0.82	−0.68	10
Legislation, polices and governance	0.10	0.84	−0.74	11

**Matrix Table 1:** Four driver matrix quadrants based on manageability and negative health consequences scores of drivers of change.

Driver matrix		Manageability of driver	
		Low	High
Negative health Consequences	Low	**Monitor and adapt** Monitor the associated hazards and create contingency plans to adapt, if new emerging risks are identified	**Analyze and optimize** Implement strategies to manage negative health consequences of associated food safety hazards
	High	**Strategize and endure** Build adaptive capabilities for short term consequences of the driver and collaborate on new management strategies for long term solutions	**Leverage and innovate** Innovate with new technologies to leverage existing strengths of the driver and improve resource allocation for effective management of associated hazards.

The matrix has four quadrants based on i) negative health consequences defined by the scores for the criteria likelihood and severity of associated emerging hazards, and ii) manageability, defined by the scores on the criteria controllability and volatility of the driver. The PROMETHEE analysis and the unicriterion netflows (i.e. netflows per criteria) per driver inform the driver matrix. When a driver of change is associated with high scores on severity and likelihood of food safety hazards, it is considered to be of high negative health consequences. When the volatility of the driver is low and its controllability high, the driver is considered highly manageable. Specifically, volatility unicriterion netflows scores lower than 0 and controllability unicriterion netflow scores higher than 0, indicate a driver which is highly manageable. If unicriterion netflow scores on likelihood and severity are higher than 0, negative health consequences are high. In cases where the categorization is not immediately obvious (e.g. likelihood is low <0 and severity is high >0), the average utility over the two criteria is taken to determine which category the driver would fall into.

## 3 Results

### 3.1 General survey results

#### 3.1.1 Criteria weights

The experts’ distribution of 100 points over the four criteria resulted in the following relative weights: 16.7 for volatility, 23.3 for controllability, 27.7 for likelihood, and 32.2 for severity criterion. The criterion severity of hazards associated with the driver is thus considered almost twice as important as the criterion volatility of the driver.

#### 3.1.2 General critical ranking

The relative importance weights of the four criteria in combination with the scores per criterion for each driver, as expressed by the experts, resulted in the overall ranking of drivers (Table 1). Ranking is based on the netflows derived from the PROMETHEE analysis (Table 1), based on the expert responses from the survey. It is clear from Table 1 that the driver with the perceived highest rank (i.e., most pressing on the food system in terms of negative health consequences and lack of manageability) is the environmental driver ‘environmental contamination’. The driver with the lowest rank is the political driver ‘legislation, polices and governance’.

### 3.2 Sensitivity analyses

#### 3.2.1 Ranking of drivers per subset of experts

The ranking of drivers per expert's employment type is presented in Table 2. For the company subset and government subset the highest ranked driver is ‘environmental contamination’, which is the same as when all employment types are combined (Table 1). The sequence of the remaining drivers is different between the two subsets, for instance, ‘food processing technologies’ is the 2nd lowest ranked driver for the company subset, and it is in 7th place for government employed respondents. While, for research institute respondents, the driver ‘food processing technologies’ is considered of lowest rank and the driver ‘geopolitical instability’ of highest rank, for academics ‘environmental contamination’ and ‘geopolitical conflict’ were found to be of the highest rank. Many of the drivers remain in a similar rank in comparison with Table 1, indicating relative robust study results.

**Table 2:** Net flow results based on the responses of the four subsets related to employment type. Rank indicated between brackets and the rank where all respondents are considered is listed in the first column.

Rank original	Drivers	Company (n = 8)		Government (n = 13)		Research Institute (n = 11)		Academia (n = 16)	
**1**	Environmental Contamination	0.77	(1)	0.43	(3)	0.54	(2)	0.66	(1)
**2**	Geopolitical instability	0.37	(3)	0.74	(1)	0.66	(1)	0.41	(3)
**3**	Bioprocesses	0.45	(2)	0.55	(2)	0.47	(3)	0.59	(2)
**4**	Management of natural resources	0.36	(4)	−0.15	(8)	−0.36	(10)	0.30	(4)
**5**	Supply Chain	0.09	(6)	−0.19	(9)	0.24	(4)	0.07	(6)
**6**	Demographic development	−0.08	(7)	0.03	(6)	−0.16	(6)	0.13	(5)
**7**	Consumer behavior	−0.11	(8)	0.30	(4)	−0.12	(5)	−0.23	(7)
**8**	Health and Wellbeing	0.2	(5)	0.06	(5)	−0.34	(9)	−0.29	(8)
**9**	Food processing technologies	−0.7	(10)	−0.15	(8)	−0.46	(11)	−0.65	(11)
**10**	Technologies in food production	−0.59	(9)	−0.72	(10)	−0.29	(8)	−0.62	(10)
**11**	Legislation polices and governance	−0.74	(11)	−0.90	(11)	−0.17	(7)	−0.37	(9)

#### 3.2.2 Stability analysis

**Fig. 1:**
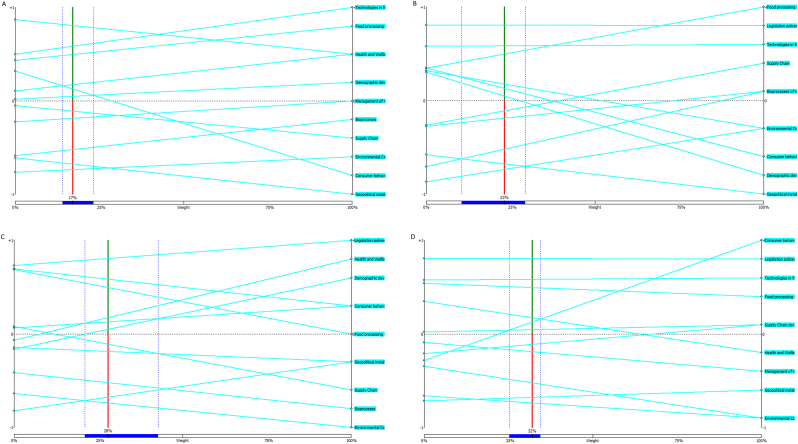
Visual stability plot, where the weight of the four criteria is varied from 0 to 100 and the netflows of each driver with each corresponding new weight is plotted. Plot A is for the criteria volatility, plot B for controllability, plot C for likelihood and plot D for severity. The vertical solid lines in each plot are the original weights defined by the expert respondents and the dashed horizonal line represents netflow 0, every criterion above has a positive netflow and every criteria below a negative netflow. The dark blue area on the horizontal axis represents the zone where the rank stays the same around the expert scored criteria weight. For volatility this expert criteria weight is: 16.7, for controllability this is: 23.3, for likelihood this is: 27.7, and for severity the expert criteria weight is: 32.2.

An important factor affecting the netflows is the weight of the criteria. Fig. 1 shows a visual stability plot depicting how a change in the weight of one criterion affects the netflows of the drivers and therefore the critical ranking. Many of the lines representing the drivers stay parallel to each other and the intersections are few, meaning the ranking stays the same. In hazard likelihood we see many intersections occurring, yet there is a large range where the rank stays the same as when using the weights scored by experts, represented by the dark blue area on the horizontal axis (between percentages 21 and 43). The stability frame for the other criteria are: volatility (14, 23), controllability (11, 29), and severity (25–35). We see large variations in the ranking when the criteria weight is above 50 %. As respondents are asked to distribute 100 points over the four criteria, it is possible, but unlikely that one criterion would receive such a high average weight. This again shows the relative robustness of the results and the critical ranking. Controllability is the criteria with the most stable ranking, which can be seen in the many parallel lines, while likelihood has many intersections indicating a fickler ranking.

### 3.3 Driver matrix

Matrix Table 2 shows the driver of change categorization in the driver matrix. The quadrant of the driver, (i.e. the location in the driver matrix) is based on the unicriterion netflows for each driver of change. The matrix categorizes the drivers of change to better support decision and policy makers. Opportunities are particularly available for highly manageable drivers, especially ‘management of natural resources’, ‘bioprocesses’ and ‘supply chain’ where the negative health consequences are high. Strategize and endure drivers are of great importance to the food system, however, they present difficulties for decision and policy makers as they have limited influence on the driver.

**Matrix Table 2:** Categorization of drivers of change into the four driver matrix quadrants.

Driver matrix		Manageability	
		Low	High
Negative health consequences	Low	**Monitor and adapt** Consumer behavior Health and well-being Demographic development	**Analyze and optimize** Legislation, policies and governance Technologies in food production Food processing technologies
	High	**Strategize and endure** Environmental contamination Geopolitical instability	**Leverage and innovate** Management of natural resources Bioprocesses Supply chain

## 4 Discussion

### 4.1 Overview

In this study drivers of change are categorized based on an MCA expert survey capturing the manageability of the driver and negative health consequences of associated hazards.

The expert survey yields a limited number of full responses. While over 100 responses are recorded, 51 are complete. Nevertheless, the sensitivity analysis shows robustness in the results. With the current responses, the results are relatively robust in the ranking. Furthermore, for a questionnaire, 51 responses are good amount to have. The surveys that were not completed did not continue past the weighting questions, likely due to the length of the survey becoming clear at that point. No relation between the drop out survey responses is evident. Additionally, the experts are spread with relatively even number of responses for risk expertise, chemical and biological food safety expertise, and their employment, which also reduces outliers or the chance certain groups overshadow others. This is crucial for an MCA where conflicting aspects are evaluated with various stakeholder groups or, in this case, various food safety expert groups (FAO, 2017; Garre et al., 2020; Ruzante et al., 2017). In this study, the drivers of change are not independent from one another and they each can influence multiple emerging risks at once. In the end, MCA is a tool to support decision makers, amongst which for food research (Fazil et al., 2008; Poissant et al., 2023; Perchec-Merien and Esguerra, 2024; Yeak et al., 2024). The current results are a guideline for decision makers to allocate time, effort and other resources to the highest critical ranked drivers. By categorizing the drivers and identifying critical driver ranking, these resources can be allocated to the most important and manageable drivers and not spent on a single driver which might be difficult to influence (Garre et al., 2020).

With the four criteria considered in this study, four different driver quadrants are established. Most drivers clearly fell into one such category. Drivers ‘health and well-being’ and ‘supply chain’ have less clear quadrant allocation. The severity of associated hazards for ‘health and well-being’ is above 0 (0.2), however the likelihood is very low (−0.8). By averaging both, it was considered to have low negative health risks. ‘Supply chain’ likelihood of associated hazard is high (0.6), however severity is low (−0.1), in a similar manner to ‘health and well-being’ it was chosen to consider both numbers and categorizing ‘supply chain’ as high negative health consequences.

In this MCA, eleven drivers of change are considered by experts, climate change or environmental change is not among them. Climate change is recognized as a significant driver of emerging food safety risks (EFSA 2020). Changing weather patterns, rising sea levels and increased frequency of extreme weather events affect all aspects of the food chain and its safety (Duchenne-Moutien and Neetoo, 2021). It has systemic and broad impact across all STEEP dimensions and simultaneously affect multiple components of the food system (FAO, 2020). The influence of climate change on food safety can overshadow other drivers of change in a research such as this one. Therefore, the climate change is classified as “mega trend” or an overarching super driver. In future research where single hazards are taken in consideration, climate change should be explicitly integrated into the analysis to capture the specific influence on the considered hazard.

### 4.2 Driver matrix

The categorizing of the drivers in the driver matrix shows the distinct quadrant allocation of each driver. This gives an opportunity for tailored driver approach and corresponding food risk management. For drivers categorized as “monitor and adapt”, limited interventions can be done as their manageability is low. The drivers in this quadrant are: consumer behavior, health and wellbeing, and demographic development. The negative health consequences are low as well and therefore regular assessments should be performed to monitor if the driver does not evolve to a critical challenge (Liu et al., 2022). Management should focus on early warning systems in order to adapt and react (Marvin et al., 2022).

The “analyze and optimize” quadrant contains drivers of change which are relatively under control, these drivers typically currently have management priority. Drivers in this quadrant are legislation, policies and governance, technologies in food production, and food processing technologies. Overall, the focus should be on incremental changes and innovation (da Cunha, 2021). Such innovations could use existing strengths to improve efficiency, cost reduction or improve sustainability (Avgoustaki and Xydis, 2020; Vågsholm et al., 2020). Examples are reprocessing surplus food, improved food safety training and sensor usage in production. Drivers that fall in this category can be beneficial for the food system, for instance, ‘legislation, policies and governance’. This driver has far reaching consequences and can shape a whole industry (Chen and Xie, 2019). For example, Bouzembrak and Marvin (2019) found that maximum residue level and the annual agricultural budget of a country, amongst others, contributed most to the predicted presence of food safety hazards in fruits and vegetables. Both maximum residue level and the agricultural budget are decided and directly influenced by legislation, policies and governance. Furthermore, the maximum residue limits in place are enforced, further incentivizing food business operators to adhere to the legislation and policies, making their managing effect on the food system even stronger. Besides the maximum limits for the presence of food safety hazards in foods, harmonizing international standards can promote innovation and stable management.

Drivers in the quadrant with more negative health consequences and high manageability, “leverage and innovate”, are management of natural resources, bioprocesses, and supply chain. For the drivers in this quadrant, it is important to have decisive mitigation action to limit food safety risks arising. Furthermore, proactive management could be implemented to prevent risks from becoming severe (Aiyar and Pingali, 2020). There are opportunities for improvement with innovation and new technologies, which could become competitive advantages for food business operators (Ahmad et al., 2023; Jin et al., 2020; Oriekhoe et al., 2024). An example is the driver supply chain. A critical aspect in the food supply chain is traceability when a food hazard contamination occurs. Blockchain technology can improve traceability by generating a transparent, detailed, precise and immutable record of batches or food items. Furthermore, efficiency is important in supply chains, this can also improve with block chain technology, for example in cases of recalls or by integrating with other smart technologies (Pandey et al., 2022; Rana et al., 2021). From policy makers adoption subsidies could be created to increase uptake of new technologies and research and development grants to promote innovation.

Finally, drivers of change within the “strategize and endure” quadrant have low management priority but have high negative health consequences and pressure on the food system (Misiou and Koutsoumanis, 2022). Both these drivers ‘environmental contamination’ and ‘geopolitical instability’ in this quadrant can hardly be managed. For food safety and production, the environment is of considerable importance, as evidenced by the great number of research articles being published (Jiang et al., 2021). Environmental contamination, where pollutants such as persistent organic pollutants and nano plastic enter the environment, can have high negative health consequences and is often a complex multifaceted challenge (Lu et al., 2015). At the same time, food production can cause much of the environmental burden and even pollution, in the forms of greenhouse gas emissions or wastewater pollution (Jiang et al., 2021; Tubiello et al., 2021). These issues are not easy to resolve; more than 20 years ago critical limitations for environmental science to solve pollution have already been identified (Huesemann, 2001). The contamination of the environment continues to this day, with many diverse negative effects without effective mitigation strategies (Sullivan et al., 2021; Yadav et al., 2021). Therefore, for the drivers in the quadrant “strategize and endure”, the food system should build adaptive capacities and become more resilient (Mu et al., 2021; Wu, 2024). This could be done via collaborative efforts, policies interventions, long term planning, technological innovation and creating redundancies (Garcia et al., 2020; Karanth et al., 2023).

### 4.3 Limitations and evidence

Differences between experts are highlighted in Tables 2 and in some cases the drivers rank very differently when compared to the ranking of all complete surveys. One such case is the governmental experts rank “consumer behavior” at number four, while in the general ranking it is number 8, which is where the governmental experts place “management of natural resources” and “technologies in food processing”. Likely, geographic differences can also play a role in the responses of experts. Most experts are either a member of the European union or working for a European company (68.6 %) and results can therefore be most relevant to Europe and other western countries. Other more accurate geographic information was not collected to anonymity protection.

To better understand these differences a workshop or Delphi study, rather than an online survey, could give insights in how they arise (Diamond et al., 2014; Schober and Conrad, 1997). The Delphi and workshop expert elicitation methods allow for interaction between experts (either anonymous or non anonymous), so in case one expert would consider the driver environment as important, other experts could adopt this as well in their opinion. However, in this study, an individual online survey was used to capture independent expert opinions, which allows the comparison between expert groups and practicality for the respondents, considering the length of the survey. Another technique to further compliment this analysis, is an approach taken by Han et al. (2024), where big data is used to support decision makers with the nuance between management options. This is only possible when sufficient high quality data is available.

The sensitivity analysis, in light of criteria weight changes, indicates that study results for the ranking of drivers of change are robust. Even with large changes in the weight of a criterion, much of the ranking remains the same and most of the drivers remain within a relative ranking region.

## 5 Conclusion

This study categorized and ranked drivers of change affecting the emergence of known food safety hazards using an expert survey based on MCA. Four different quadrants for drivers of change are identified: monitor and adapt, analyze and optimize, leverage and innovate, and strategize and endure. The matrix quadrant “strategize and endure” captures drivers that are critical (: ‘environmental contamination’ and ‘geopolitical instability’) whereas the quadrant leverage and innovate captures drivers that are of high health consequences but also manageable. The quadrants based on MCA unicriterion netflows can assist decision and policy makers in the food system in regard to food safety, and pave the way for more effective resource allocation and management strategies in the future.

## CRediT authorship contribution statement

**N.M.C. Hommels:** Conceptualization, Methodology, Validation, Formal analysis, Investigation, Writing – original draft, Writing – review & editing, Visualization. **M.C.M. Mourits:** Conceptualization, Methodology, Writing – review & editing, Supervision. **M. Focker:** Conceptualization, Writing – review & editing, Supervision, Funding acquisition. **H.J. van der Fels-Klerx:** Conceptualization, Resources, Writing – review & editing, Supervision, Funding acquisition.

## Funding

This research has received funding from the European Union's 10.13039/100018693Horizon Europe Research and Innovation Program under Grant Agreement No. 101060698. Additional funding from the 10.13039/501100013890Ministry of Agriculture, Nature and Food Quality through project No KB-37-002-023 is acknowledged.

## Declaration of competing interest

The authors declare the following financial interests/personal relationships which may be considered as potential competing interests: Marlous Focker reports financial support was provided by 10.13039/501100013890Ministry of Agriculture, Nature and Food Quality. Nina Hommels reports financial support was provided by 10.13039/501100000780European Union 10.13039/100019637Horizon Research and Innovation Program. If there are other authors, they declare that they have no known competing financial interests or personal relationships that could have appeared to influence the work reported in this paper.

## Data Availability

Data will be made available on request.
